# Editorial: Activation of Innate Immunity by Allergens and Allergenic Sources

**DOI:** 10.3389/falgy.2021.800929

**Published:** 2021-11-12

**Authors:** Fatima Ferreira, Geoffrey A. Mueller, Stefanie Gilles, Marsha Wills-Karp

**Affiliations:** ^1^Department of Biosciences, Paris-Lodron University of Salzburg, Salzburg, Austria; ^2^Department of Health and Human Services, Genome Integrity and Structural Biology Laboratory, National Institute of Environmental Health Sciences, National Institutes of Health, Research Triangle Park, NC, United States; ^3^Department of Environmental Medicine, Faculty of Medicine, University of Augsburg, Augsburg, Germany; ^4^Institute of Environmental Medicine, Helmholtz Center Munich-Research for Environmental Health, Augsburg, Germany; ^5^Department of Environmental Health and Engineering, Johns Hopkins Bloomberg School of Public Health, Baltimore, MD, United States

**Keywords:** beta-glucosylceramide, dendritic cell, epithelial barrier, mast cell, sphingolipid, Toll like receptor signaling, type 2 innate immunity

Allergic inflammation is a type 2 immune disorder characterized by upregulation of interleukin-4 (IL-4), IL-5, IL-9, and IL-13, which are collectively referred to as type 2 cytokines, by the development of Th2 cells and the production of high levels of allergen-specific IgE antibodies (1). However, the stimuli and mechanisms influencing the initiation of the type 2 innate immune responses remain unclear and are presently intensively investigated (2–7). Typically, macrophages, group 2 innate lymphoid cells (ILC2s), neutrophils, mast cells (MCs), eosinophils, basophils, as well as associated non-immune cells (e.g., epithelial cells) are involved in type 2 innate immunity by providing an environment that supports the subsequent activation of adaptive immunity. Activation of such innate cells is mainly mediated by pattern-recognition receptors (PRRs) (e.g., Toll-like receptors, TLRs; C-type lectin receptors, CLRs; NOD-like receptors, NLRs), which form the sensory interface of the innate immune system (8). Both, the allergen themselves and danger signals, which are present in the allergen source or in the environment, have been shown to directly activate PRRs (9–11). Pattern-recognition receptor activation on dendritic cells (DCs) may directly promote the induction of inflammatory TH2 cells. Pattern-recognition receptors are also expressed on epithelial cells and their activation leads to secretion of thymic stromal lymphopoietin (TSLP), GM-CSF, and distinct cytokines, which can indirectly trigger DC activation followed by Th2 induction.

The first evidence for the involvement of PRRs in allergic sensitization came from the observation that Der p 2 could act as a functional mimic of myeloid differentiation factor 2 (MD-2), the lipopolysaccharide (LPS)-binding component of the TLR-4 signaling complex. Der p 2-LPS complex could activate TLR4 signaling in MD-2-deficient mice and in the presence of MD-2, Der p 2 facilitated LPS-dependent signaling, thus demonstrating its auto-adjuvant activity (12, 13). Interestingly, the structural fold of cockroach allergen Bla g 1 was shown to bind various lipids including lipoteichoic acid, which binds specifically to TLR2 (14, 15). Uptake by carbohydrate receptors (e.g., mannose receptor, MR; DC-SIGN), a mechanism exemplified by the glycan-dependent capacity of Ara h 1 to bind DC-SIGN and to induce Th2 differentiation of naïve T cells, has also been proposed (16, 17).

The present research topic compiles seven contributions addressing the role of innate immunity in shaping adaptive immune responses to various allergens. Collectively, they support the view that diverse stimuli, receptors, and mechanisms are involved in the initiation of type 2 innate immunity by allergen sources (11, 18, 19).

The article by Buelow et al. focused on the function of *Alternaria alternata* (Alt) in initiation of peanut allergy in neonatal mice with heterozygous mutations in genes encoding the skin barrier proteins filaggrin and mattrin [flaky tail mice (FT^+/−^)]. These mice exhibit oral peanut-induced anaphylaxis after skin co- exposure to Alt, peanut extract (PNE), and detergent. The authors demonstrated that Alt stimulated an increase in the expression of IL-33, a cytokine that functions as an alarmin and plays important roles in type 2 innate immunity via activation of eosinophils, basophils, MCs, macrophages, and ILC2s. In addition, Alt stimulated a pathway involving oncostatin M and amphiregulin, which acted together with IL-33 and PNE to induce food allergy in mice with skin barrier mutations but not in wild type neonates.

Sphingolipids and their derivatives (e.g., sphingosine-1-phosphate, ceramide-1-phosphate, b-glucosylceramide) are involved in many cellular processes and play important roles in regulating cell biology and organismal homeostasis by promoting cell survival, migration, and differentiation. Moreover, sphingolipids were shown to be important players in immunity due to modulatory effects on the cytokine profile of innate and adaptive immune cells. The review by Dias-Perales et al. presents an overview on the role of sphingolipids in the development of allergic sensitization and allergic inflammation via the activation of immune cells resident in tissues and their role in barrier remodeling and anaphylaxis. The authors discussed how alterations in the levels of sphingolipids and their receptors contribute to immune dysregulation and the promotion of allergic sensitization. Hence, the authors proposed that binding of sphingolipids to allergen molecules such as the interaction between phytosphingosine and Pru p 3, the peach LTP (20) may play an important role in allergic sensitization.

The study by Walker et al. focused on the identity of maternal factors of allergic mothers that alter offspring responsiveness to allergen. Lipids are known to be altered during allergic responses and are transported to the fetus for growth and formation of fetal membranes. Thus, the authors hypothesized that pro-inflammatory lipids are elevated in allergic mothers, are transported to the fetus and regulate fetal immune development. Indeed, allergic mothers showed a significant two-fold increase in β-glucosylceramides (βGlcCer), which was transported across the placenta from the mother's plasma to the fetus and in breastmilk to the offspring. Importantly, administration of βGlcCer to non-allergic mothers was sufficient for offspring responses to allergen. In addition, maternal administration of a pharmacological inhibitor of βGlcCer synthase normalized the levels of βGlcCer in the allergic mothers and her offspring and blocked the offspring allergen responsiveness. The identification of βGlcCer as a risk factor induced by allergen exposure in allergic mothers is an important novel insight into our understanding of environment-host interactions and risk factors for allergic disease early in life.

Two comprehensive reviews by Jacquet and by Keshavarz et al. summarized current knowledge of innate immune responses to house dust mites (HDM) and their allergens. Jacquet focused on the discussion of identified obstacles and limitations in this research area. The author commented on difficulties due to (i) the presence of various non-allergenic intrinsic (mite exoskeleton) and extrinsic (environmental and endosymbiotic microorganisms, pollutants) immunostimulators, (ii) the degree of purity of allergen preparations, (iii) correct folding of recombinantly produced HDM allergens, (iv) limited availability and intrinsic variability of human epithelial samples used in the experiments, and (v) insufficient translation of data obtained in mouse models to humans. This detailed review also identified the areas where further research is needed to address essential knowledge gaps and highlighted the importance of establishing the clinical relevance of innate immune pathways in HDM allergy.

Keshavarz et al. presented a different perspective in their review, which focused on the role of innate immunity in shaping adaptive responses to HDM and ticks. Tick bites were shown to induce a form of delayed allergy to mammalian meat mediated by IgE antibodies to the oligosaccharide galactose-a.1,3-galactose (a-Gal) (21). Despite the fact that sensitization to HDM fecal particles occurs predominantly via the respiratory tract and tick bites via the skin, two salient features are common to both allergenic sources: (i) the delivery of multiple PAMPs/DAMPs into a local area of the skin or mucosa and (ii) epithelial disruption activity. The authors extensively discussed the role and contributions of identified factors and molecules from HDM fecal particles and tick saliva to allergic immunity. Despite substantial progress in this area, more information on source-specific Th2 promoting PAMP/DAMPs, their interaction with host cells and on induced host factors is needed for understanding the origins and development of allergic immunity.

In the study presented by Pointner et al., the role of TLR4 in DC activation by birch pollen extract (BPE) and its relevance in the initiation of adaptive immune responses was examined. The activation Murine and human DCs induced by either BPE or LPS was inhibited by the TLR4 antagonist or by polymyxin B (PMB), and abrogated in murine TLR4-deficient bone marrow-derived DCs (BMDCs) compared to wild-type BMDCs. *In vivo*, immunization with BPE induced a significant Th2 polarization, whereas administration of BPE pre-incubated with PMB showed a decreased tendency. These findings suggest that TLR4 is a major pathway by which BPE triggers DC activation that is involved in the initiation of adaptive immune responses. However, the exact nature of the BP-derived TLR4 adjuvant remains unclear and should be addressed in future studies.

Finally, Srivastava and Kaplan summarize recent advances in transcription factors (TFs) involved in the development of MCs throughout the body and in specific tissues, as well as those involved in responses to extracellular milieu stimuli. Though MCs are known as the main effector cells in IgE-mediated allergic reactions, they also play an important role in innate immunity. Mast cells recognize pathogens and are activated during innate immune responses by multiple mechanisms including TLRs and receptors for complement (22). The authors provided a detailed description of TF networks affecting MC functions during allergic inflammation, cell degranulation, and cytokine secretion. In addition, the authors highlighted the need of further studies focusing on the mechanism regulating TF networks as well as on the role of chromatin and chromatin modifying proteins in MC from various tissue sites. Further insights in these areas are needed for better understanding the tissue-specific and pro-allergic functions of MCs.

## Author Contributions

FF designed and wrote the article. GM, SG, and MW-K equally designed the article, read, and made comments on the manuscript. All authors contributed to the article and approved the submitted version.

## Conflict of Interest

The authors declare that the research was conducted in the absence of any commercial or financial relationships that could be construed as a potential conflict of interest.

## Publisher's Note

All claims expressed in this article are solely those of the authors and do not necessarily represent those of their affiliated organizations, or those of the publisher, the editors and the reviewers. Any product that may be evaluated in this article, or claim that may be made by its manufacturer, is not guaranteed or endorsed by the publisher.
